# Ridge Augmentation Is a Prerequisite for Successful Implant Placement: A Literature Review

**DOI:** 10.7759/cureus.20872

**Published:** 2022-01-02

**Authors:** Anilkumar R, Rekha R Koduganti, Tata Sai Lakshmi Harika, Haripriya Rajaram

**Affiliations:** 1 Department of Periodontics, Panineeya Mahavidhyalaya Institute of Dental Sciences & Research Centre, Hyderabad, IND

**Keywords:** inlay onlay and inter-positional grafting, maxillary sinus lift, nerve transportation, ridge expansion, ridge augmentation

## Abstract

Alveolar ridge defects are commonly seen in partially dentate patients, and they jeopardize speech, appearance, and oral hygiene maintenance. These defects affect both soft tissues and bone and are mainly caused by trauma. These defects are more prevalent in middle-aged male patients and it is imperative that the defective ridge is augmented before receiving an implant or a fixed partial denture. This review focuses on the different types of ridge defects and their treatment options.

## Introduction and background

Globally, fixed restorations are increasingly being replaced by dental implants and these are preferred by both patients and clinicians alike [1]. The increasing popularity of implant dentistry is due to the high success rate of nearly 95% in both edentulous and partially edentulous conditions [2]. However, the long-lasting success of implant placement depends upon sufficient density and volume of the bone available [3,4]. Often, patients present insufficiencies in relation to ridge width and height; moreover, they may also have an unfavorable soft tissue profile [5,6]. To treat these insufficiencies, a multitude of agents and techniques are available. However, there is a lack of consensus related to the success of these surgical techniques [7-9]. This article aims to review the currently available ridge augmentation procedures supported by related studies and also highlights the emerging trends and future directions in this field. To facilitate this, we searched the electronic databases ScienceDirect, PubMed, Scopus, and Google Scholar to obtain relevant data. A combination of related keywords was used, such as "Ridge Augmentation", "Ridge Expansion", Nerve Transportation", "Maxillary Sinus Lift", "Inlay", "Onlay", and "Interpositional Grafting". Full-text, relevant articles published from 1980 to 2021 in dental journals, including case-control studies, cross-sectional studies, and systematic and short reviews, were analyzed.

## Review

Hard tissue augmentation procedures

The technique applied to treat these ridge defects varies based on the severity of involvement. Width-associated defects can be treated with autologous grafts and membranes; however, height-related ridge deficiencies are more complex and may require onlay grafting. The various procedures performed to treat the horizontal ridge defects include ridge split, ridge expansion, and sandwich osteotomy combined with sinus floor grafting for severe alveolar atrophy. The faciolingual bone density could be improved by placing graft and membrane after decortication. Also, block grafts from intraoral or extraoral sources could be placed to augment the ridge width. For ridge height defects, distraction osteogenesis or block graft with a membrane is the usual norm.

Role of Barrier Membranes in Ridge Augmentation

Barrier membranes, when used to augment the bone, follow the guided bone regeneration (GBR) principle and are available in both non-resorbable and resorbable forms. The main task of GBR is to aid in protecting the clot and preventing epithelial and connective tissue cells from migrating into the healing wound, thereby favoring the cells of osteogenic lineage to populate the area. The membrane should be biocompatible, cell-occlusive, should allow space maintenance, and should have good handling properties, with optimal degradation time. For the GBR technique to be successful, it should provide primary wound closure, angiogenesis, clot organization, and prevention of dead space. A membrane collapse will cause fibrous growth preventing osteogenesis. Adequate space under the membrane to allow the migration of cells and the ingrowth of new blood vessels is thus mandatory. Larger defects may require support with particulate bone and possibly titanium mesh.

Rüedi and Bassett [10] and Boyne [11] used Millipore filters in their studies on long bones in the 1960s. However, they did not prove to be significant in popularizing the barrier membrane application in patients. Saravanan et al. studied the potential of autografts, xenografts, and resorbable collagen membranes in 10 patients with horizontal ridge deficiencies. Using CT, bone width was evaluated at 2 mm, 4 mm, and 6 mm from the alveolar crest preoperatively and 180 days postop. An improvement in ridge width at 2 mm (3.63 ±0.29 to 5.07 ±0.25), 4 mm (4.10 ±0.18 to 5.51 ±0.21), and 6 mm (4.47 ±0.25 to 5.88 ±0.27) was found after 180 days [12].

Meloni et al. conducted a cohort prospective study to evaluate the regeneration of osteoid in 45 patients with horizontal bone defects. A 1:1 mixture of particulate bovine bone graft and autogenous graft was used in layers to fill the defects, over which the membrane was placed and secured. Prosthetic loading in tapered body implants was done after six months. The implant success was 100% during the follow-up period and CBCT performed at the initial examination and seven months postop revealed a gain in the crestal bone [13].

Another study was performed on 18 patients who required implants and had deficient ridges. Both auto and xenograft were introduced in a particulate form in a 1:1 proportion, with the injectable form of platelet-rich fibrin (i-PRF) and absorbable collagen membrane covering the regenerated region and leukocyte PRF (L-PRF) membrane covering the GBR membrane in the horizontal defect. The same graft materials were also employed for the height-deficient bone, but it was protected by a titanium-reinforced polytetrafluoroethylene membrane covered by L-PRF. The results pointed to a bone gain in width and height [14].

Alveolar Distraction Osteogenesis (ADO)

This was the most frequently performed technique in craniomaxillofacial surgery between 1954 and 1971. Gavriel Ilizarov developed this novel surgical approach for skeletal deformities. It follows the principle of "tension-stress" with slowly incorporated tensile stress promoting histogenesis. Bone traction generates tension and promotes osteogenesis, which occurs parallel to the distraction site. The distraction of the segment can be in vertical and/or a horizontal direction. Distractor devices are of an intraosseous or extraosseous configuration. Intraosseous devices are generally introduced into the transport segment, making the precise introduction of the devices more critical. Adequate bony thickness is crucial with most intraosseous devices, e.g., Leibinger Endosseous Alveolar Distraction (LEAD) implant system. Extraosseous devices are generally more feasible to place because they are applied laterally onto the bone, but they may complicate patient comfort and aesthetics, e.g., track-plus systems. The indications are as follows: there should be a clearance of 6-7 mm from vital anatomical structures. Phases of distraction osteogenesis include phase I or surgical phase, phase II of latency, phase III or distraction phase, and finally phase IV or consolidation phase. A distractor is fixed between the transported and basal bone segments keeping a distance of 1-2 mm and left in place for a week to allow for the formation of a callus. The activation of the distractor is then initiated at 0.5-2 mm/day. After the distraction has been achieved to the required magnitude, the distractor is removed and the bone formed is qualitatively assessed. Additional grafts may have to be placed before the placement of implants. The main advantage of employing this technique is that soft tissue grafting is either avoided or minimized; moreover, the distraction device can be maintained and activated by the patients at home. The drawback is the additional cost of the device and difficulty in controlling the vector of distraction.

Some researchers have evaluated the complications that arose during mandibular alveolar distraction osteogenesis and suggested treatments for these. In a study by Garcia et al., a total of seven distractions were done in five patients by using an intraosseous distractor. Complications occurred in all the cases; however, they were minor and could be averted using an appropriate technique [15].

In another study, two devices (implant device and orthodontic screw) were applied on 28 patients to facilitate the movement in the transverse and longitudinal planes. The researchers of this five-year study observed a movement of 6.5 mm in the vertical sector whereas only a 2-mm movement was seen in the horizontal sector. They concluded that the maxilla has better results with vertical distraction procedures [16].

Almarrawi conducted a study to evaluate the role of horizontal ADO and the fate of implants placed in the distracted bones of 12 healthy individuals with decreased ridge width in the mandibular posterior region. Horizontal ADO was performed to treat the lost parts. Implants were placed after four months and assessed. They showed a 100% implant success rate before loading and 94.1% after loading. Thus, the author concluded that ADO can be a reliable surgical method to correct ridge deficits [17].

Onlay Grafting

Inadequate palatal vault morphology is caused by excessive bone resorption. Onlay grafts are indicated in these situations, and they could be either particulate onlay grafting or block onlay grafting. Particulate onlay grafting in turn is subdivided into direct particulate onlay grafting and subperiosteal tunnel grafting. Saddle depressions and horizontal and vertical ridge defects are the apt indications for direct onlay grafting.

Direct particulate onlay grafting: this technique involves raising a full-thickness flap at the affected site and giving releasing incisions to aid in tension-free adaptation. After the flap is elevated, the bone is drilled with holes to aid in the retention of the graft material and ensure good osseointegration. Defects that are non-contained require that both graft and membrane are placed to cover them and thus promote the treatment outcome. Demineralized graft materials are the first choice and their retention can be extended by using tissue adhesives or protein-based regenerative gels.

Cordaro et al. studied 22 ridges with horizontal deficiency having two terminal teeth working as abutments. The control group (11 ridges) received multiple mandibular bone blocks to treat the deficient ridges whereas in the test group (11 ridges), in addition to the blocks being inserted at the periphery, deproteinized bovine bone mineral was given, covered by a collagen membrane. Four months later, implants were installed and it was observed that the bone resorption was lower in the test group [18].

In another study, 16 bone grafts from the retromolar area were utilized on 11 patients. CBCT was used to assess the treatment outcome. It was observed that there was a 43.7% resorption of the grafted bone. This valid point should be taken into account before the implant is placed [19].

Subperiosteal tunnel grafting: this is a procedure performed for the deficiency of the ridge width rather than the height. The technique performed is as follows: after anesthetizing the area, an incision is given away from the affected site and a subperiosteal tunnel is created from the access incision by using tunneling instruments after which the demineralized graft is injected into the tunnel. Digital manipulation may be required to ascertain the correct placement of the graft. Then the incised area is sutured. This procedure is associated with minimal graft exposure or dehiscence. Some clinicians used the subperiosteal tunneling approach for lateral ridge augmentation in seven patients. Bovine grafts were placed, followed by implant placement after four months, which showed good stability [20].

Hamed et al. compared minimally invasive horizontal ridge augmentation with subperiosteal tunneling versus open flap technique in 10 patients with horizontal ridge defects. Six months postop, it was observed that the healing was better in the minimally invasive subperiosteal tunneling group [21].

Block Onlay Grafting

This procedure is performed for either class I or class II ridge defects and sometimes also for class III ridge defects (Siebert’s Classification). The recipient site is prepared initially by reflecting a full-thickness flap and drilling many holes till the spongiosa; then, based on the severity of the ridge defect, the onlay graft is selected. An inverted J block graft is used for vertical defects, whereas a veneer graft is used for horizontal defects. For class III defects, either inverted J graft or a lamellar technique of graft placement is preferred. A lamellar technique involves a veneer graft placement in the cortical area and the placement of particulate graft underneath it to cover the defect. This graft is then immobilized with screws.

Schwartz-Arad and Levin described alveolar ridges augmentation using intraoral block onlay graft in 10 patients before implantation and they concluded that this grafting procedure shows good success in long-span augmentation [22]. Another study evaluated the success of autogenous block graft procured from ascending part of mandibular ramus for augmenting the mandibular posterior region laterally. A 10-year follow-up was done on 24 patients who underwent 39 lateral ridge augmentation procedures and 48 implant placements, showing good treatment outcomes [23].

Interpositional Bone Graft

This procedure is indicated for vertical ridge defects with alveolar dimensions of 4-5 mm wherein there is no soft tissue deficit. The idea is to place two different layers of bone grafts and then cover them with a barrier membrane, creating a structure like the cross-section of the bone. After placing a vestibular incision and elevating the flap initially, vertical corticotomy is performed at the mesial and distal end of the recipient site followed by a horizontal corticotomy at the vestibular region. This will enable a block of bone to be moved coronally. This block graft is then fixed to the basal bone by means of mini plates, and the gap created by lifting the block graft is filled with cortico-cancellous autograft. Care is taken to avoid impingement into vital anatomical structures. The elevated flap is then sutured back.

A study was performed on eight individuals to evaluate the stability of ridge augmentation for the restoration of implants using interpositional bone graft. Radiographic analysis was done at the grafted site, which showed good stability of implants [24]. Another study was conducted in the anterior maxilla for the assessments of vertical ridge augmentation using the onlay and inlay bone grafting technique in 16 patients who were to receive 40 implants. CBCT assessment was done pre and postoperatively, which showed a bone gain in the inlay bone grafting group [25].

Ridge Split Technique

Tatum developed specific instruments including tapered channel formers and D-shaped osteotomes to expand the resorbed residual ridges of both the upper and lower jaws having a ridge width of <3 mm. The procedure is initiated by reflecting a mucoperiosteal flap, after which a crestal incision is given; then a vertical osteotomy (10-12 mm) is performed on both the terminal portions of the deficient ridge by taking care to avoid damaging the adjacent teeth. Horizontal ridge split is done using conventional burs or piezosurgical instruments. After osteotomy is complete, the facial and lingual walls are spread apart by using osteotomes to make space for the placement of the implant. The gap between the implant and bone is then filled with particulate graft material over which a membrane is placed and stabilized. After four to six months, the implants are loaded.

Moro et al. have given a description of specially designed new tips for treating patients with a transversal bone deficit. The study included 15 patients and the tips that were used were very safe and effective [26]. Another study evaluated the outcome of the ridge split technique among 60 patients. Mid-crestal incision and the piezoelectric system were used for bone management. The implant was placed in expanded alveolar bone and the gap between implant and bone was filled with autologous bone and xenograft, which led to 97.5% of osseointegration around the implants [27].

Ridge Expansion

This is indicated in patients with ridge width <6 mm. A full-thickness flap is raised to expose the bone. Leaving a gap of 1 mm away from the abutment teeth on both sides, a horizontal osteotomy is done encompassing the length of the implants that are to be given. Then an osteotomy in the vertical plane is made at the distal-most point of the horizontal osteotomy on both sides. Osteotomy holes are then prepared with a pilot drill (1.2-2 mm). Expansion screws with an expansion limit of 2.5 mm are then introduced to expand the bone. This technique facilitates bone expansion by 1 mm.

Brugnami et al. have presented a technique by combining tapered bone expander and piezoelectric scalpel to expand the ridge for implant placement, The authors showed that it has high patient acceptability and is technically very simple for surgeons to perform [28]. Another study included 26 partially edentulous sites, with a one-stage alveolar ridge augmentation procedure using titanium mesh along with GBR and immediate implant placement. CBCT was used for the evaluation of bone gain. It showed a significant increase in buccolingual bone width without any gain in alveolar crestal bone height.

Inferior Alveolar Nerve Transportation

Anatomical structures like the inferior alveolar nerve (IAN) are often jeopardized when placing implants, especially if it courses very close to the apices of teeth. To prevent this catastrophe, a procedure called IAN transportation is performed [29].

Barbu et al. have presented a surgically modified technique for repositioning IAN on seven patients with severely atrophic mandibles. This procedure prevented the loss of keratinized tissue around the crestal area, and also enabled a safe and easy placement of implants [30].

Chehata and Abdelmonim conducted a comparative clinical study between piezosurgery and conventional rotary instruments in IAN transportation, which was followed by the placement of implants. They observed that conventional surgical burs caused high postoperative pain, neurosensory dysfunction, and edema compared to piezosurgical instruments [31].

Sinus Lift Procedures for Vertical Ridge Augmentation

The posterior maxillary region often poses a problem regarding the placement of dental implants owing to the pneumatization of the maxillary sinus from tooth loss due to caries or periodontal disease. Tatum [32] proposed a technique, "sinus lift procedure", for implant placement when there is insufficient bone between the maxillary alveolus and sinus. Alveolar height <10 mm is often an indication for sinus lift surgery via the crestal (indirect) approach, while alveolar height <5 mm via is an indication for the lateral (direct) approach. Contraindications for sinus lift procedures include poor oral hygiene, severe parafunctional habits, dental infection, drug or alcohol abuse, untreated acute or chronic sinusitis, use of anticoagulants or antiplatelet medications, neoplasms, local cysts, and radiation therapy. Lateral window technique (lateral or direct sinus lift) and crestal approach (crestal or indirect sinus lift) are the two procedures of sinus lift available.

Indirect sinus lift technique: the crestal or indirect sinus lift is indicated when the residual bone height is less than 6-8 mm. After LA is given, a pilot or initial drill is used, followed by drills in increasing diameters, and the osteotomy site is prepared. Care is taken to ensure that the drill length is maintained at 2 mm away from the floor of the sinus. As drills of higher diameter are introduced, it is observed that the sinus floor gets fractured and the sinus is slowly elevated to avoid injury to the Schneiderian membrane, by using a surgical mallet/osteotome with controlled force. If required, autogenous graft material is inserted within the socket.

Direct sinus augmentation technique: This is indicated when the residual alveolar bone (RAB) is 5 mm or less. A full-thickness flap is raised giving a crestal incision and a vertical releasing incision. The bone is exposed and sometimes a bluish hue is seen on the bony surface, which is indicative of the sinus. Then a window is made either using bure or piezosurgical instruments to delineate the sinus. After the window is prepared, it is slowly disengaged to expose the sinus. Care is taken to avoid perforation of the Schniederian membrane that lines the sinus. The sinus is then slowly elevated using the appropriate sinus lift instruments. If the window created has not been totally disengaged, it could now be placed below the relocated sinus to form its floor. The empty void created between the elevated sinus and the basal bone is filled with either autologous or allogeneic graft material and a membrane is stabilized over it. Boyne and James et al. issued the first publication on this technique by using autogenous grafts deposited into the sinus and allowing it to be healed by six months.

Khandelwal et al. evaluated a one-stage direct sinus lift procedure in 13 individuals with atrophic posterior maxillae and the results showed a gain in bone width and height around the implants postoperatively with a 96.3% success rate [33]. Another study was conducted on 20 patients who needed a sinus lift for simultaneous implant placement; the patients were split into two groups: group I involving Nanobone and PRF and group II involving Nanobone and PRF with simvastatin. Group II showed superior bone fill and density when compared to group I [34].

Orthodontic Extrusion

In this method, forces are applied to the periodontally hopeless teeth, which will bring the alveolar bone along with it. Elongation of the tooth in its alveolus causes shifting of gingival and periodontal ligament (PDL) fibers. The result is a coronal shift of bone at the base of the defect as the tooth moves coronally.

Salama and Salama have documented clinical cases employing forced eruption on hopeless teeth to augment bony tissues in implant sites and also proposed a classification for extraction socket according to their morphology and placement of the implant into the socket [35]. Arun and Shreemogana (2018) stated that the PDL cells play a crucial role at a molecular level, thereby aiding in optimal results after implant placement [36].

Soft tissue augmentation procedures

According to Berglundh and Lindhe, the "biologic width" of an implant is the minimum amount of mucosal attachment required to maintain implant stability. The thickness of the mucosa could be improved by using connective tissue grafts (CTG). Soft tissue deformities in partially edentulous patients could be treated by different techniques that have shown good results. These procedures include pedicle graft procedure, roll flap, modified roll technique, free graft procedure, pouch graft procedure, interpositional graft procedure, and onlay graft procedure.

Roll Flap Procedures

This was proposed by Abrams [37] and is indicated in small-to-moderate class I ridge defects, primarily in cases with a single-tooth space. This procedure was the first among the periodontal surgical procedures that have been developed to augment ridge deformities. The basic concept of the procedure entails the creation of a pedicle of connective tissue that is placed or tucked into a subepithelial pouch. After achieving profound local anesthesia, a rectangular pedicle flap is created on the palatal aspect of the defect. The flap dimension should tally with the amount of soft tissue required at the recipient site. It must be noted that if there are two or three pontic spaces to be treated, separate pedicles are raised. A sharp Bard-Parker blade is used to remove the epithelium over the palatal flap. A pouch is now created in the supra-periosteal connective tissue on the labial aspect. The palatal pedicle graft, which is de-epithelized, is now rolled back and tucked into position labially. Sutures are placed to secure the rolled flap in its position. The de-epithelized area heals by secondary intention.

Mali et al. have described the roll flap procedure as an alternative to restore the damaged ridge with varying success [38]. Padhye et al. compared the subepithelial connective tissue graft (SCTG) and buccally displaced flap, and the results showed an increase in the width and thickness of keratinized mucosa in the buccally displaced flap group than the SCTG group, with reduced surgical sites, less postop pain, and good blood supply [39].

Modified Roll Technique

This technique is a modification of the roll technique wherein the epithelium over the connective tissue is not scraped but preserved to cover the donor site. It was introduced by Scharf and Tarnow [40], in class I deformities. A Bard-Parker blade is used to make an incision from the crest to the palatal area, taking care to include a sufficient length of the tissue to be rolled to the desired area on the buccal aspect. A similar incision is made on the other side to include sufficient width of the graft, and then the two vertical incisions are connected by a horizontal incision. A partial-thickness trap door-type flap is reflected. The pedicle is rolled on the buccal aspect and stabilized using a horizontal mattress suture. A review by Kinaia et al. has described the beneficial use of the modified rolled CTG technique for improving the peri-implant soft tissues [41].

A few clinicians have reported that the modified roll technique can be used for selected mild to moderate class I ridges, resulting in uneventful healing and good esthetic outcome [42].

Pouch Graft Procedure

Some researchers have developed a method of managing ridges with horizontal loss of dimensions. In this procedure, a subepithelial pouch is created in the defective area and a CTG harvested from the palate is placed. Indications for this procedure include class I defects, converting a thin gingival biotype into a thick biotype, and correction of soft tissue defects around restored implants or non-submerged implants. Local anesthesia is given and the initial incision is made. After a pouch is created, the harvested CTG is placed into the pouch in a buccocoronal direction to increase the soft tissue dimensions [43].

A study by Agarwal and Gupta compared the treatment outcomes of modified CTG and SCTG in a pouch in 40 patients who were divided into two groups. Patients in group I received modified CTG whereas group II received SCTG in the pouch. The authors observed that group I patients showed better results related to soft tissue thickness when compared to group II [44].

Another study by Gupta et al. has reported that the pouch method is ideal for fixed prosthodontic treatment as it improves the aesthetics, phonetics, and also reduces food impaction in the pontic region [45].

Interpositional Graft Procedure

This technique involves the placement of graft without scraping the epithelium from the connective tissue when both the buccolingual and apicocoronal augmentation of the soft tissue is required. An envelope partial-thickness flap at the facial surface is prepared. A provisional bridge is placed to estimate the dimensions of the graft to be harvested. A periodontal probe is used to measure the length, width, and depth of the void of the pouch, and a suitable donor site is selected in the palate or the tuberosity area, and a free graft of epithelium-connective tissue is excised, which is then transferred and placed in position at the recipient site. To improve the buccolingual contour, the epithelium of the graft harvested is placed flush with the surrounding epithelium; however, if both the buccolingual and apicocoronal aspects of the recipient site have to be augmented, then the graft is sutured slightly more coronal to the surrounding tissue. The swelling due to inflammation helps in sculpturing the contour of the ridge below the provisional bridge. Eventually, after healing, a smooth surface of the epithelium is achieved.

Shetty et al. have studied a case of a localized ridge defect in a 37-year-old male patient in relation to the right maxillary canine and premolar region. The defect was a Siebert’s class III defect and was treated with an onlay interpositional graft harvested from the palate. A fixed partial restoration was given later with a good treatment outcome [46]. Another case, involving a 40-year-old female patient with a Siebert’s class I ridge defect in the maxillary anterior region, was managed using a combination of roll flap procedure and onlay interpositional graft. After seven weeks, it was observed that the emergence profile improved remarkably and the patient was given a full ceramic restoration later [47].

Onlay Graft Procedure

This procedure was first described by Siebert [48] and is indicated in class II ridge defects wherein the vertical height of the ridge is defective. The technique involves the placement of anesthetic solution high in the vestibular fornix and in the palate, thereby obtaining vasoconstriction in the recipient site. A scalpel is used to remove the epithelium with short, saw-like strokes at the recipient site at the level of 1 mm below the outer surface. The margins can be prepared with either a butt joint or a beveled margin. The harvested graft should be a few millimeters longer and wider than the dimensions required. The graft is transferred with tissue forceps for a try-in and is trimmed to the proper shape and adjusted to fit the connective tissue surface of the prepared ridge. The recipient bed and lateral borders are prepared and made raw to facilitate a good blood supply to the graft when placed.

A case report by Desai et al. has analyzed a defective maxillary anterior ridge that was treated using a free gingival autograft, which showed good improvement in aesthetics postoperatively [49]. Another study was conducted on five patients with defective ridges wherein CTG, keratinized gingival graft, and pediculated CTG were used and follow-up was done for 5-30 years. All the grafts placed showed good outcomes and stability [50].

## Conclusions

In this review, we engaged in a discussion of the various ridge augmentation procedures and studies related to their treatment outcomes. Currently, a major focus of periodontal research is on the role of growth factors in bone and tissue regeneration. Recombinant growth factors have been shown to accelerate the healing of dermal and bone tissues. Emerging technologies include growth factors like fibroblast growth factors (FGFs), insulin-like growth factors (IGFs), and bone morphogenic proteins (BMPs). Platelet-derived growth factor (PDGDF) alpha and beta receptors have shown good regenerative potential in PDL cells as well as osteoblasts. For intraoral applications, BMP 2 and 7 have been shown to give good results. The regenerative response could be promoted using localized gene therapy wherein the concentration of growth factors is increased. Scaffolds to deliver cells and genes include prefabricated solid as well as injectable forms that can be introduced into periodontal defects. 3D-printed scaffolds have revolutionized regenerative periodontal therapy as they are able to be customized to suit the defect. Thus, these biologicals could help promote wound healing at a molecular level and could prove to be a boon in the field of periodontics.
